# Gastroenteritis Outbreaks Caused by a DS-1–like G1P[8] Rotavirus Strain, Japan, 2012–2013

**DOI:** 10.3201/eid2006.131326

**Published:** 2014-06

**Authors:** Seiji P. Yamamoto, Atsushi Kaida, Hideyuki Kubo, Nobuhiro Iritani

**Affiliations:** Osaka City Institute of Public Health and Environmental Sciences, Osaka, Japan

**Keywords:** genetic constellation, genome constellation, G1P[8]I2, G1-P[8]-I2, DS-1–like G1P[8], young children, Osaka, Japan, viruses, rotavirus, gastroenteritis, outbreaks, epidemics

## Abstract

Rotavirus A (RVA) genotype G1P[8], a hallmark of the Wa-like strain, typically contains only genotype 1 genes. However, an unusual RVA G1P[8] with genotype 2 genes was recently detected in Japan. We determined the complete genomic constellation of this RVA. Our findings suggest that mixed RVAs may be more competitive than once thought.

It is estimated that rotavirus A (RVA), the leading cause of severe gastroenteritis in children worldwide, causes >500,000 deaths among children each year, primarily in developing countries (*1*). Genes of viral protein (VP) 7 and VP4 form the basis of a dual classification system that defines the RVA G- and P-types, respectively. Five G-types (G1–4 and G9) and 3 P-types (P[4], P[6], and P[8]) represent most of the G-P–combined RVA strains (*2*). RVAs are classified on the basis of a system that assigns a specific type to each of the 11 RNA gene segments, according to established nucleotide percentage cutoff values (*3*).

Two well-known RVA prototype strains are Wa (G1-P[8]-I1-R1-C1-M1-A1-N1-T1-E1-H1) and DS-1 (G2-P[4]-I2-R2-C2-M2-A2-N2-T2-E2-H2). RVAs G1P[8], G3P[8], G4P[8], and G9P[8] are pure Wa genogroup members because they have a Wa-like constellation (Gx-P[x]-I1-R1-C1-M1-A1-N1-T1-E1-H1) composed of genotype 1 genes; G2P[4] is a pure DS-1 genogroup member because it has a DS-1–like constellation (Gx-P[x]-I2-R2-C2-M2-A2-N2-T2-E2-H2) composed of genotype 2 genes (*4*). The segmented nature of RVA genomes enables them to undergo gene reassortment during co-infection in 1 cell, leading to the emergence of progeny viruses containing mixed segments from >2 different parental strains. However, some human RVA G/P-types have a purely Wa-like or a DS-1–like genome constellation. Mixed viruses are rarely detected and have a low prevalence, even if they emerge; thus, it is believed that mixed viruses may be less fit than parental strains and unable to compete with them (*5*).

We identified and characterized a prevalent genotype G1P[8] RVA with genotype 2 genes. The RVA was detected during rotavirus gastroenteritis outbreaks in Japan. 

## The Study

During 2009–2013, a total of 21 RVA-associated gastroenteritis outbreaks occurred among children ≤7 years of age in Osaka City, the third largest city in Japan; 20 of the outbreaks were in nursery schools and 1 was in a primary school (Table 1). To determine which outbreaks were associated with RVA, we tested fecal samples from children who became ill during the outbreaks. An RVA-associated outbreak was defined as a gastroenteritis (vomiting and/or diarrhea) outbreak among >8 children who were epidemiologically linked, among whom >2 had RVA-positive ELISA results by our testing. Analyses to determine epidemiologic links included age, outbreak setting, person-to-person virus transmission, and date of symptom onset. 

**Table 1:** Description of RVA–associated outbreaks in Osaka City, Japan, during 2009–2013*

Outbreak year, month	Outbreak no.	No. patients	No. specimens collected/no. positive for RVA	RVA genotype
2009				
April	HC09022	24	6 (2)	G9-P[8]-I1
2010				
April	HC10032†	19	1 (1)	G1-P[8]-I1
December	HC10062	Unknown	2 (2)	G2-P[4]-I2
2011				
April	HC11027	20	3 (2)	G1-P[8]-I1
May	HC11030	18	3 (2)	G9-P[8]-I1
2012				
March	HC12010	18	3 (3)	G1-P[8]-I1
April	HC12012	26	5 (3)	G1-P[8]-I2
April	HC12013	41	5 (2)	G1-P[8]-I2
April	HC12014†	15	5 (1)	G9-P[8]-I1
April	HC12016†	18	3 (1)	G1-P[8]-I2
April	HC12018†	23	3 (1)	G1-P[8]-I2
April	HC12020	32	2 (2)	G1-P[8]-I2
May	HC12021	29	3 (2)	G1-P[8]-I1
May	HC12022	18	2 (2)	G1-P[8]-I2
2013				
March	HC13027	33	5 (4)	G1-P[8]-I1
May	HC13037	26	5 (2)	G1-P[8]-I2
May	HC13040	8	2 (2)	G1-P[8]-I2
May	HC13041†	26	1 (1)	G1-P[8]-I2
May	HC13043	21	5 (2)	G1-P[8]-I2
May	HC13048	11	2 (2)	G1-P[8]-I2
May	HC13049	15	5 (3)	G1-P[8]-I2

*All outbreaks occurred in nursery schools, except outbreak no. HC10062 (Dec 2010), which occurred in a primary school. RVA, rotavirus A. †Only 1 sample had test results positive for RVA but the outbreak was confirmed on the basis of epidemiologic data from hospital clinics showing that other patients had RVA–positive test results.

We extracted viral RNA from RVA-positive 10% fecal suspensions and converted the RNA to cDNA by using SuperScript III Reverse Transcriptase (Thermo Fisher Scientific Inc., Waltham, MA, USA) and RVA-specific primers (*6*). PCR was performed with Ex Taq DNA Polymerase (TaKaRa Bio Inc., Shiga, Japan); the products were used for direct sequencing with a BigDye Terminator Cycle Sequencing Kit (Thermo Fisher Scientific). RotaC v2.0 was used for RVA genotyping (*7*), and nucleotide alignment was confirmed by using ClustalW (http://www.clustal.org/) in MEGA5.2 (*8*). MEGA5.2 was used to identify the optimal evolutionary model that best fit each sequence dataset and to construct maximum-likelihood trees (*8*).

All detected RVA strains were sequenced and genotyped for 3 regions: VP7 (877 bp), including antigenic epitopes 7-1a, 7-1b, and 7-2 (*9*); VP4 (656 bp), including antigenic epitopes 8-1 to 8-4 (*10*); and VP6 (1,132 bp). Only 1 RVA genotype was detected in each outbreak (Table 1). G1-P[8]-I1, the most common Wa-like genotype worldwide (*4*), was detected in a few outbreaks each year during 2010–2013. However, an unusual RVA genotype, G1-P[8]-I2, first identified in April 2012, was detected in 6 (66.7%) of the 9 RVA outbreaks in 2012 alone. Moreover, infections caused by genotype G1-P[8]-I2 were detected in 6 (85.7%) of the 7 RVA outbreaks in 2013. Phylogenetic analysis based on the VP7, VP4, and VP6 regions showed that 12 genotype G1-P[8]-I2 strains were closely related to each other (>99% nt identity) (Figure). These data suggest that G1-P[8]-I2 originated from 1 parental strain and became the prevalent genotype in RVA-associated outbreaks in Osaka City during 2012–2013.

**Figure:**
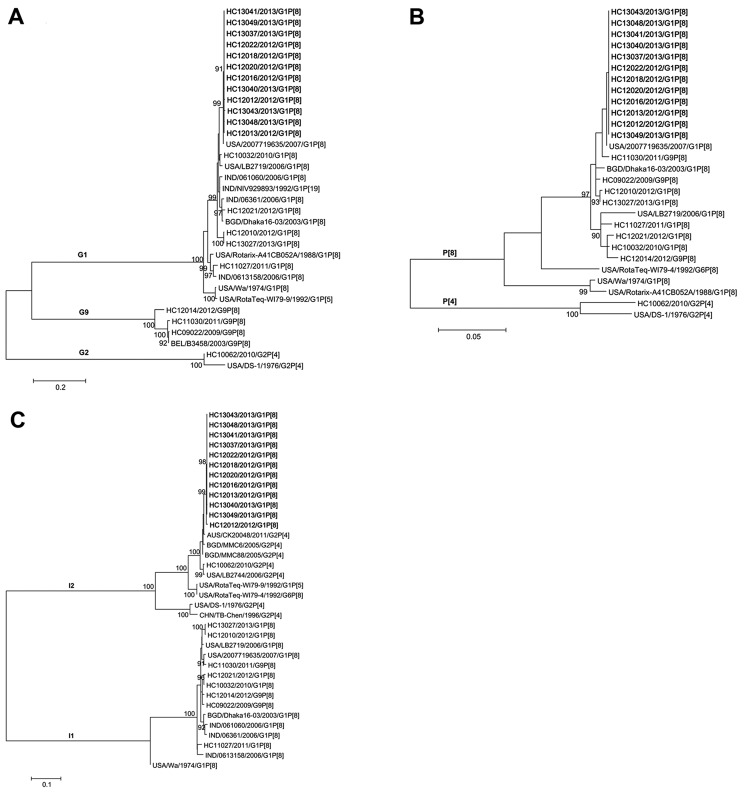
Maximum-likelihood phylograms of the viral protein (VP) 7 (877 bp) (A), VP4 (656 bp) (B), and VP6 (1,132 bp) (C) regions of rotavirus A strains detected during outbreaks in Osaka City, Japan, 2009–2013. The strain names are associated with outbreak numbers listed in Table 1. Boldface font indicates G1-P[8]-I2 strains. On the basis of Akaike information criteria, with a correction for finite sample sizes, general time reversible plus gamma (+G) plus invariable sites (+I), Tamura 3-parameter +G, and Tamura 3-parameter +G +I models were used for genes VP7, VP4, and VP6, respectively. Numbers at nodes indicate the bootstrap support values, given as a percentage of 1,000 replicates (values <90 are omitted). Scale bars indicate genetic distances.

We selected 6 strains for study. The strains had been isolated during outbreaks HC12012, HC12016, HC12022, each of which occurred at the beginning, middle, or end of an RVA G1-P[8]-I2 outbreak period in 2012, and during outbreaks HC13037, HC13043, and HC13049, each of which occurred at the beginning, middle, or end of an RVA G1-P[8]-I2 outbreak period in 2013. We determined the complete genome constellations of the 6 strains on the basis of partial sequences of the other 8 RNA gene segments. Genotyping of all 11 RNA gene segments showed that the 6 strains each had 9 genotype 2 genes: G1-P[8]-I2-R2-C2-M2-A2-N2-T2-E2-H2 (DS-1–like G1P[8]). As a representative of DS-1–like G1P[8] strains, HC12016/2012/G1P[8] (hereafter referred to as strain HC12016) was used for analysis of nearly full-length sequences of all 11 RNA gene segments. Strain HC12016 (listed in Table 2) was deposited in the DNA Data Bank of Japan as RVA/Human-wt/JPN/HC12016/2012/G1P[8] under accession nos. AB848004–AB848014. 

**Table 2:** Nucleotide sequence identity of various RVA strains to RVA DS-1–like G1P[8] strain HC12016, which was detected during an outbreak in Osaka City, Japan, 2012*

Strain name	Genogroup	% Nucleotide identity by gene segment										
		VP7	VP4	VP6	VP1	VP2	VP3	NSP1	NSP2	NSP3	NSP4	NSP5
Wa†	Wa	91.96	90.30	79.73	79.94	80.92	77.14	75.14	81.59	78.83	81.19	81.69
2007719635‡	Wa	99.60	99.44	79.73	80.07	80.41	77.14	75.16	82.88	78.62	80.41	84.29
DS-1§	DS-1	72.56	87.22	87.68	90.17	93.93	93.06	92.60	85.93	94.65	89.34	92.91
CK20048¶	DS-1	72.96	87.09	98.82	99.25	99.30	87.82	99.02	99.26	97.38	98.28	99.24
CK20030#	DS-1	72.76	87.43	98.74	99.41	97.77	87.70	99.09	98.84	97.59	98.28	97.13
CK20038**	DS-1	75.18	87.09	96.00	96.78	99.41	98.66	96.65	97.57	99.27	88.71	98.94
PA130††	DS-1	73.24	87.26	98.59	98.97	97.53	87.53	98.60	98.62	97.27	98.43	97.13
Okayama strains‡‡	DS-1	99.78–100	99.90–100	99.84–100							98.75–99.69	99.70–100

*Strain HC12016 is RVA/Human-wt/JPN/HC12016/2012/G1P[8] (G1-P[8]-I2-R2-C2-M2-A2-N2-T2-E2-H2). The various strains included in this table are genetically related to strain HC12016, and of the strains tested, these showed the highest sequence identity to strain HC12016 in each gene segment. Underlining indicates the highest identity to strain HC12016, excluding DS-1–like G1P[8] strains. Dark gray indicates >99% identity to strain HC12016, excluding DS-1–like G1P[8] strains. Light gray indicates >98% identity to strain HC12016, excluding DS-1–like G1P[8] strains. RVA, rotavirus A; VP, viral protein; NSP, nonstructural protein. †RVA/Human-tc/USA/Wa/1974/G1P[8] (G1-P[8]-I1-R1-C1-M1-A1-N1-T1-E1-H1). ‡RVA/Human-wt/USA/2007719635/2007/G1P[8] (G1-P[8]-I1-R1-C1-M1-A1-N1-T1-E1-H1). §RVA/Human-tc/USA/DS-1/1976/G2P[4] (G2-P[4]-I2-R2-C2-M2-A2-N2-T2-E2-H2). ¶RVA/Human-wt/AUS/CK20048/2011/G2P[4] (G2-P[4]-I2-R2-C2-M2-A2-N2-T2-E2-H2). #RVA/Human-wt/AUS/CK20030/2006/G2P[4] (G2-P[4]-I2-R2-C2-M2-A2-N2-T2-E2-H2). **RVA/Human-wt/AUS/CK20038/2008/G6P[4] (G6-P[4]-I2-R2-C2-M2-A2-N2-T2-E2-H2). ††RVA/Human-wt/ITA/PA130/2010/G2P[4] (G2-P[4]-I2-R2-C2-M2-A2-N2-T2-E2-H2). ‡‡RVA/Human-wt/JPN/OH3385/2012/G1P[8], RVA/Human-wt/JPN/OH3493/2012/G1P[8], RVA/Human-wt/JPN/OH3506/2012/G1P[8], and RVA/Human-wt/JPN/OH3625/2012/G1P[8] (G1-P[8]-I2-Rx-Cx-Mx-Ax-Nx-Tx-E2-H2), which were detected in Okayama Prefecture, Japan (*11*).

In addition, we performed a BLAST (http://blast.ncbi.nlm.nih.gov/Blast.cgi) search of each RNA gene segment of strain HC12016. The results showed that the VP7 and VP4 genes of RVA/Human-wt/USA/2007719635/2007/G1P[8] (hereafter referred to as strain 2007719635), a pure Wa genogroup member, shared the highest identity with strain HC12016 (Table 2). In contrast, 4 pure DS-1 genogroup strains contained >1 gene showing the highest identity, but none of those strains shared >98% identity in all 9 genes (i.e., VP6, VP1–VP3, and nonstructural protein [NSP] 1–5) (Table 2).

It should be noted that while our manuscript was under review, G1P[8] strains that had 3 genotype 2 genes (VP6, NSP4, and NSP5) were reported from a different location in Japan (Okayama Prefecture) (*11*); these strains were also called DS-1–like G1P[8]. The genes of these strains showed 98.8%–100.0% identity with strain HC12016 genes (Table 2).

## Conclusions

We determined the full genomic constellation of the DS-1–like G1P[8] strain that emerged in 2012. Our results suggest that this strain, detected in Osaka City, seems to be an intergenogroup reassortant between a G1P[8] strain (Wa genogroup) and a pure member of the DS-1 genogroup. Five genes (VP7, VP4, VP6, NSP4, and NSP5) of the DS-1–like G1P[8] strains from Osaka City were closely related to those of DS-1–like G1P[8] strains from Okayama Prefecture (*11*), indicating that the strains may have originated from the same parental strain. DS-1–like G1P[8] was prevalent in Osaka City and Okayama Prefecture around the same time (*11*); thus, because of its rapid spread, the strain should be monitored further. 

The extent of rotavirus vaccination in Japan has varied, so the level of vaccine coverage in the country is unknown. Thus, it is unclear whether the emergence of strain DS-1–like G1P[8] was caused by vaccine pressure. The introduction of rotavirus vaccines must be followed by full genomic analysis of RVA so that reassortants between wild-type and vaccine strains can be monitored. Such analyses may also lead to the discovery of new wild-type reassortants with unusual genome constellations and provide us with plausible explanations for rotavirus epidemics.

It is unclear why some RVA G/P-types have pure genogroup genomic constellations (*5*). Although the whole VP7 and VP4 amino acid sequences of strains HC12016 and 2007719635 are identical (data not shown), strain HC12016 contains genes that encode DS-1–like proteins. In contrast, strain 2007719635 is a pure Wa genogroup member, which suggests that G1P[8] type outer capsid proteins encoded by strain HC12016 are compatible with Wa-like and DS-1–like backbones. In other words, the DS-1–like G1P[8] seems to retain its fitness, even though it is the progeny of a reassortment event between viruses of different genogroups. Further studies using DS-1–like G1P[8] strains may provide key insights into preferred combinations of RVA genes derived from different genotypes.
